# Targeting microbiome-driven epigenetic modifications: a new frontier in breast cancer treatment

**DOI:** 10.1186/s13148-025-02046-0

**Published:** 2026-01-25

**Authors:** Yuhan Bai, Xiangyi Kong, Jing Wang

**Affiliations:** Department of Breast Surgical Oncology, National Cancer Centre/National Clinical Research Centre for Cancer/Cancer Hospital, Chinese Academy of Medical Sciences and Peking Union Medical College, Beijing, China

**Keywords:** Breast cancer, Microbiome, Epigenetic regulation, SCFAs

## Abstract

Breast cancer remains a leading cause of morbidity and mortality among women worldwide, with significant heterogeneity in its development and treatment response. Recent advances in understanding the roles of the microbiome and epigenetic regulation have opened new avenues for addressing the complexities of breast cancer progression and therapeutic resistance. This review explores the intricate relationship between the gut and intratumoral microbiomes and epigenetic modifications, such as DNA methylation, histone modifications, and non-coding RNAs. Specifically, we examine how microbial metabolites, particularly short-chain fatty acids (SCFAs), regulate gene expression via epigenetic mechanisms, influencing tumor growth, metastasis, and treatment response. The impact of metabolic diseases, including obesity and type 2 diabetes mellitus (T2DM), on breast cancer risk through microbiome-mediated epigenetic changes is also discussed. Furthermore, the review highlights emerging therapeutic strategies that integrate microbiome modulation with epigenetic therapies, including the use of probiotics, dietary interventions, and fecal microbiota transplantation (FMT), as well as DNA methyltransferase (DNMT) inhibitors and histone deacetylase (HDAC) inhibitors. These innovative approaches hold promise for overcoming treatment resistance and improving clinical outcomes in breast cancer patients. Future research should focus on elucidating the molecular pathways through which the microbiome influences epigenetic regulation and developing personalized, microbiome-targeted therapies that enhance the efficacy of existing treatments. By targeting both the genetic and epigenetic drivers of breast cancer, microbiome-based interventions represent a novel frontier in the fight against this challenging disease.

## Highlights

1. Summarized the epigenetic regulatory effects of microorganisms and their metabolites on the occurrence, development, and treatment of breast cancer.
2. Summarized the impact of dietary substances and their interaction with gut microbiota on the epigenetic regulation of breast cancer.
3. Discussed the relationship between metabolic diseases, microorganisms, and breast cancer at the epigenetic regulatory level.

## Introduction

Breast cancer is the most commonly diagnosed malignancy among women worldwide, accounting for a significant proportion of cancer-related morbidity and mortality. According to the World Health Organization (WHO), in 2022, an estimated 2.3 million women were diagnosed with breast cancer, resulting in approximately 685,000 deaths globally. Breast cancer incidence is higher in developed nations due to lifestyle and screening, while mortality is greater in less developed regions due to healthcare disparities [1].

Breast cancer is heterogeneous with subtypes: (1) Hormone receptor-positive (ER+) tumors respond to endocrine therapy; (2) HER2-positive tumors grow aggressively but benefit from targeted therapies; (3) Triple-negative (TNBC) lacks ER, PR, and HER2, is aggressive, prevalent in young women and in women of African descent some strains of which produce colibactin, and has high recurrence [2]. Genetic mutations in BRCA1, BRCA2, and others increase risk [3]. Management faces challenges like treatment resistance, influenced by the tumor microenvironment (TME) affecting growth and response [4].

Recent research has also highlighted the role of epigenetic regulation in breast cancer progression. Epigenetic changes, such as DNA methylation, histone modifications, and non-coding RNA regulation, alter gene expression without changing the DNA sequence. These modifications can drive tumor growth, promote metastasis, and contribute to therapy resistance. Another emerging area of interest is the microbiome, particularly the role of the gut and intratumoral microbiota in influencing cancer development and response to treatment. Evidence suggests that microbial communities may impact cancer progression through metabolic and immune modulation, as well as through epigenetic alterations, further complicating the therapeutic landscape [5].

This review aims to explore the intricate relationship between the microbiome and epigenetic regulation in the context of breast cancer. Specifically, it seeks to address how these factors contribute to the initiation, progression, and treatment resistance of breast cancer. By understanding the underlying mechanisms through which the microbiome influences epigenetic modifications, this review aims to identify potential therapeutic strategies that could enhance treatment efficacy and overcome resistance in breast cancer patients. This interdisciplinary approach holds promise for the development of novel interventions that integrate microbiome modulation and epigenetic therapies in breast cancer treatment.

## Microbiome and breast cancer

### Role of the gut microbiome

The gut microbiome plays a crucial role in influencing the host's cancer progression through the production of metabolites such as short-chain fatty acids (SCFAs), which regulate tumor cell behavior. SCFAs, including acetate, propionate, and butyrate, are produced through the fermentation of dietary fibers by gut bacteria. Among these, butyrate has been shown to exert significant anti-cancer effects by inhibiting histone deacetylases (HDACs), thereby altering gene expression and promoting apoptosis in tumor cells [6]. In the context of breast cancer, several studies have identified distinct differences in the composition of the gut microbiota between breast cancer patients and healthy individuals. For instance, Porphyromonas and Peptoniphilus are found in higher abundance in the gut of breast cancer patients, whereas beneficial bacteria like Escherichia and Lactobacillus are more prevalent in individuals with benign breast lesions [7].

Furthermore, premenopausal breast cancer patients exhibit significant alterations in gut microbial diversity compared to healthy premenopausal controls. Specifically, there is a reduction in SCFA-producing bacteria and the enzymes involved in SCFA biosynthesis, which may contribute to disease progression [8]. Notably, species such as Odoribacter, Butyricimonas, and Coprococcus have been found to decline in breast cancer patients, suggesting a link between gut microbial diversity and breast cancer risk [9].

Interestingly, the gut microbiome’s influence extends to the metabolic interactions between maternal diets and offspring breast cancer susceptibility. Studies have shown that maternal consumption of n-3 polyunsaturated fatty acids (PUFA) is positively correlated with increased abundance of Bifidobacterium in offspring, which in turn reduces breast cancer risk. Conversely, maternal diets deficient in PUFA reduce beneficial microbiota and increase tumor incidence in offspring [10, 11].

### Impact of the microbiome on breast cancer prognosis

Recent studies have established that intratumoral microbiota are present in breast tumors, which often reside intracellularly within malignant and immune cells, encompass phyla such as Proteobacteria, Firmicutes and Actinobacteria [12, 13]. The functional role of intratumoral bacteria is becoming increasingly recognized, as different bacterial species may be selectively enriched based on the breast cancer subtype. For example, Turicibacter is found in higher abundance in TNBC tumors, whereas Klebsiella and Staphylococcus are more commonly associated with HER2-positive breast cancer [14]. Notably, Fusobacterium nucleatum has been detected in breast tumors and functionally linked to metastatic progression through activation of Notch/β‑catenin pathways [15]; small extracellular vesicles derived from F. nucleatum may also signal via TLR4 to potentiate tumor growth and dissemination [16]. Additionally, circulating tumor cells (CTCs) have been found to harbor intracellular bacteria, such as Lactobacillus, which can enhance cell survival during metastasis by improving resistance to fluid shear stress [17].

Building on these observations, multiple cohorts indicate that gut and intratumoral microbiome features correlate with treatment response and survival in breast cancer, likely through epigenetic reprogramming driven by microbial metabolites (e.g., SCFAs) and immune pathways. In particular, butyrate—an SCFA with context‑dependent HDAC‑modulating activity—has been shown to enhance anti‑PD‑1 efficacy by tuning CD8 + T‑cell programs [18]. By contrast, dysbiosis marked by depletion of SCFA producers is associated with inferior responses to chemotherapy/radiotherapy [19].

Furthermore, dysbiosis, characterized by an overabundance of certain bacterial species, may negatively affect patient prognosis. Bacteria such as Bacteroides uniformis, Clostridium bolteae, and Parabacteroides merdae have been associated with poorer clinical outcomes in breast cancer patients [20]. These findings highlight the potential of targeting the microbiome as an adjunctive therapy to improve breast cancer treatment efficacy and reduce therapy resistance. Additionally, probiotics and dietary interventions that promote the growth of beneficial bacteria, such as Lactobacillus and Bifidobacterium, have shown promise in preclinical models of breast cancer, where they inhibited tumor growth and metastasis [21, 22].

## Epigenetic regulation in breast cancer

### Overview of epigenetic regulatory mechanisms

Epigenetic regulation refers to heritable changes in gene expression that occur without alterations to the underlying DNA sequence. In breast cancer, multiple epigenetic mechanisms, such as DNA methylation, histone modifications and non-coding RNAs, work in concert to modulate oncogene and tumor suppressor gene activity, thereby influencing tumor development, progression and therapeutic response. These mechanisms are not merely theoretical; specific epigenetic alterations have been well documented in breast tumors.

DNA methylation typically involves the addition of a methyl group to cytosine residues in CpG islands (usually in gene promoters) by DNA methyltransferases (DNMTs), leading to gene silencing. For instance, hypermethylation of the BRCA1 promoter is observed in approximately 10–20% of sporadic breast cancers, particularly in triple-negative cases [23], and the RASSF1A tumor suppressor gene is found to be methylated in the majority of primary breast tumors [24]. Such aberrant methylation can inactivate critical tumor suppressors, while conversely, global DNA hypomethylation can activate oncogenic sequences and induce genomic instability.

**Fig. 1:**
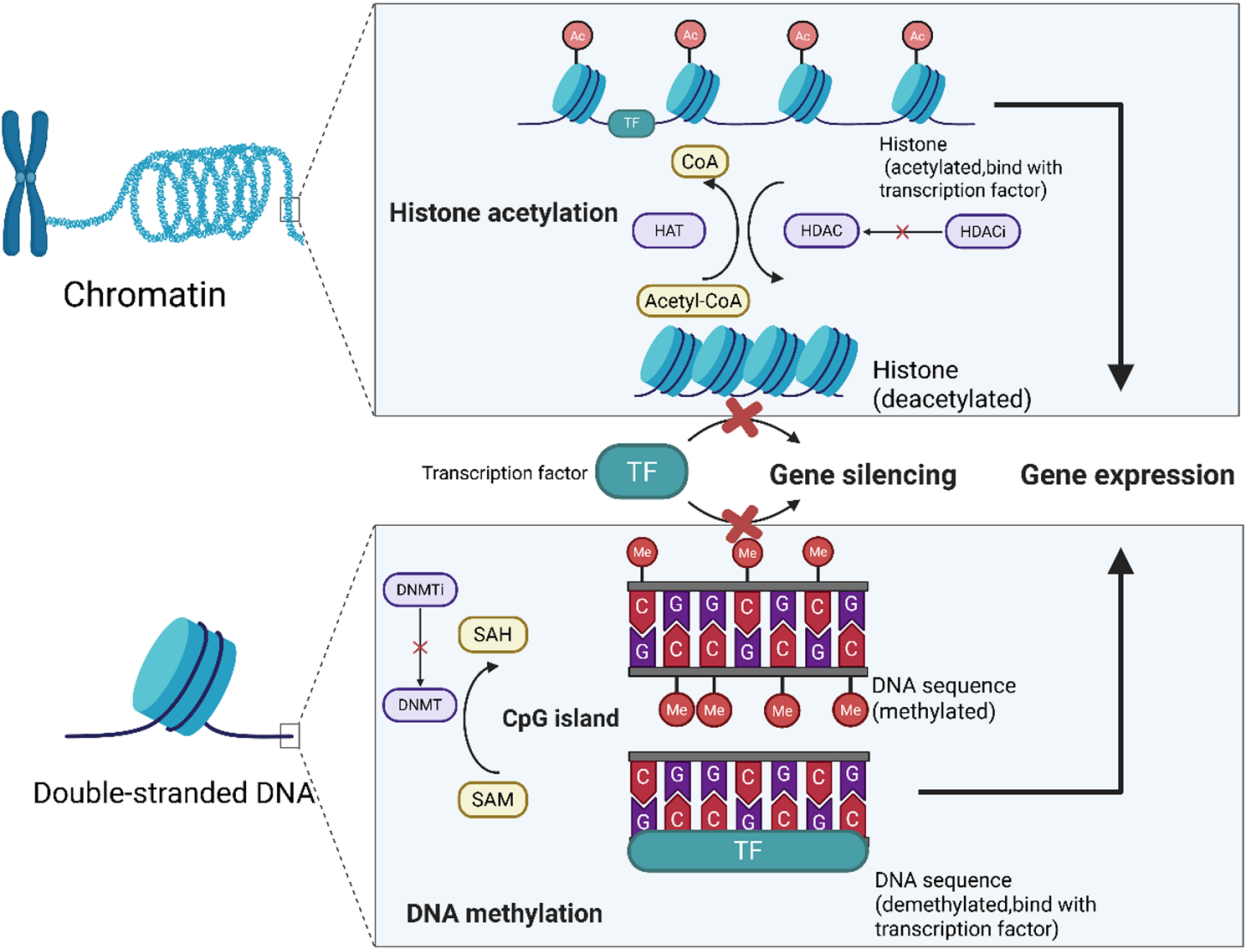
The diagram of DNA methylation and histone acetylation. DNA methylation involves the addition of methyl groups to DNA (often at CpG sites), typically leading to gene silencing. In contrast, histone acetylation (addition of acetyl groups to histone tails) relaxes chromatin structure, allowing active gene transcription (Figure created by the authors using BioRender)

Histone modifications (including acetylation, methylation, phosphorylation, and ubiquitination of histone tails) remodel chromatin structure and thus control gene accessibility. In breast cancer cells, abnormal patterns of histone marks are common; for example, overexpression of the histone methyltransferase EZH2 (catalyzing H3K27 trimethylation) leads to widespread silencing of differentiation genes and is strongly associated with aggressive tumors and poor patient prognosis [25, 26]. Meanwhile, loss of acetylation at certain histone lysines or inappropriate gain of activating marks (like H3K4me3 and H3K27ac at oncogene loci) can also dysregulate gene expression programs to favor cancer cell survival [27]. Figure 1 provides a schematic illustration of some of these mechanisms: DNA hypermethylation in gene promoters (often depicted as red lollipops on DNA) compacts chromatin and silences gene transcription, whereas histone acetylation (orange flags on histone tails) relaxes chromatin to activate transcription.

Non-coding RNAs also play a pivotal epigenetic role by regulating gene expression post-transcriptionally or by guiding chromatin modifiers to specific genes. In breast cancer, oncomiRs such as miR-21 are frequently upregulated, directly targeting tumor suppressors like PTEN and PDCD4; this disinhibition of the PI3K/AKT and other oncogenic pathways promotes cell proliferation, invasion, and metastasis [28]. Likewise, certain long non-coding RNAs are dysregulated—notably, HOTAIR is highly overexpressed in metastatic breast tumors and recruits the PRC2 complex (which deposits repressive H3K27me3 marks) to dozens of genes, reprogramming chromatin and enhancing tumor invasiveness [29].

In summary, aberrations in DNA methylation, histone modifications, and non-coding RNA expression do not act in isolation; rather, they establish an “epigenetic landscape” that drives malignant transformation. Collectively, these epigenetic alterations cooperate to rewire gene expression networks in breast cancer cells, leading to uncontrolled growth, acquisition of stem cell-like properties, and resistance to conventional therapies.

### Role of DNA methylation in breast cancer

DNA methylation changes are a hallmark of breast cancer and are intimately linked to disease behavior and therapy outcomes. Hypermethylation of tumor suppressor gene promoters such as BRCA1, PTEN, and RASSF1A is frequently observed in breast malignancies, resulting in the epigenetic silencing of these critical genes. The loss of tumor suppressor expression through promoter hypermethylation promotes cancer cell survival and proliferation. This phenomenon is especially evident in hormone receptor-positive subtypes (luminal A and luminal B), where silencing of genes involved in cell cycle regulation and DNA repair can augment estrogen-driven oncogenic signaling [30]. Conversely, global DNA hypomethylation including demethylation of normally silenced repetitive elements or oncogenes, can lead to genomic instability and activation of pro-tumorigenic pathways in breast tissue.

Clinically, DNA methylation changes have been implicated in therapy resistance. For example, hypermethylation of the ESR1 (ERα) gene promoter can downregulate ER expression, rendering estrogen-dependent tumors unresponsive to endocrine treatments like tamoxifen or aromatase inhibitors. Similarly, aberrant methylation of DNA repair genes has been linked to chemotherapy resistance, as cancer cells with silenced repair pathways may escape the lethal DNA damage intended by cytotoxic drugs. These insights have motivated the exploration of DNMT inhibitors (e.g., azacitidine, decitabine) in breast cancer. DNMT inhibitors can demethylate DNA and reactivate epigenetically silenced genes, thereby resensitizing cancer cells to treatment. Early-phase clinical studies and preclinical models have shown that DNMT inhibitors can restore the expression of tumor suppressors and potentially slow tumor growth when used alone or in combination with standard therapies. Notably, in TNBC, which lacks targeted hormonal or HER2-directed treatments, DNMT inhibitors are being investigated as an adjunct to chemotherapy to overcome resistance [31]. Recent studies also suggest that targeting DNMTs may diminish pro-survival pathways in drug-resistant breast cancer cells; for instance, DNMT inhibition can disrupt cholesterol biosynthesis (a metabolic adaptation in resistant cells) and even induce pyroptosis (inflammatory cell death), thereby enhancing the efficacy of chemotherapeutics such as paclitaxel. The ongoing research into demethylating agents underscores the therapeutic potential of reversing DNA methylation abnormalities as part of a multi-modal breast cancer treatment strategy [32–34].

### Histone modifications and breast cancer therapy

Histone modifications are another central layer of epigenetic dysregulation in breast cancer, and they offer a rich array of therapeutic targets. Aberrant histone marks in breast tumors can lead to either activation of oncogenes or repression of tumor suppressor genes. For example, inappropriate acetylation at certain histone sites (such as H3K27ac at super-enhancers) may amplify oncogene expression, while excessive methylation at others (or loss of methylation at key residues) can shut down genes that normally restrain tumor growth [35]. A prominent example is the EZH2 enzyme mentioned earlier: EZH2 is often overexpressed in aggressive breast cancers (particularly TNBC) and drives transcriptional repression of genes involved in differentiation and apoptosis [26]. Elevated EZH2 not only correlates with higher tumor grade and poor prognosis, but also helps maintain a stem-like, therapy-resistant state in cancer cells. This has led to the development of EZH2 inhibitors (such as tazemetostat) aimed at reactivating silenced genes; preclinical studies demonstrate that inhibiting EZH2 can impair TNBC cell proliferation and reduce metastatic potential [25].

Another well-validated target class is the HDACs, which remove acetyl groups from histones, compress chromatin, and generally suppress gene transcription. In breast cancer, HDACs are frequently dysregulated—for instance, HDAC2 and other Class I HDACs are found to be overexpressed or amplified in basal-like breast cancers, contributing to the epigenetic silencing of tumor suppressors. Notably, high HDAC2 expression is significantly associated with high tumor grade, positive lymph node status, and shorter survival [36]. To counteract this, HDAC inhibitors (HDACis) have been tested in breast cancer models with promising results. These agents (e.g., vorinostat, entinostat, panobinostat) prevent the removal of acetyl groups, thereby keeping chromatin in a more open, transcriptionally active state. By doing so, HDACis can reactivate silenced genes (including those encoding cell cycle inhibitors and pro-apoptotic factors) and induce growth arrest or apoptosis in breast cancer cells [37]. For example, the HDAC inhibitor mocetinostat showed potent anti-tumor effects in vitro against basal-like breast cancer cells that overexpress HDAC2, leading to increased histone acetylation and re-expression of DNA damage response genes that suppress tumor growth [36]. Early clinical trials of HDACis in advanced breast cancer have suggested potential benefits, especially when combined with other treatments, although their optimal use is still under investigation.

In addition to histone acetylation and methylation modifiers, histone demethylases are gaining attention in breast cancer therapy. Lysine-specific demethylase 1 (LSD1/KDM1A), which erases methyl marks on histone H3 (particularly H3K4me1/2), is aberrantly overexpressed in many breast cancers. LSD1 has a dual role: it can co-repress tumor suppressor genes (often partnering with HDACs) and also sustain oncogenic gene expression programs, thereby promoting proliferation, metastasis, and even endocrine therapy resistance. In luminal breast cancer models, for instance, LSD1 contributes to maintaining the epithelial–mesenchymal transition and cancer stem cell properties that underlie invasion and anti-estrogen resistance [38]. Targeting LSD1 with specific LSD1 inhibitors is therefore a promising strategy. Preclinical studies have shown that inhibiting LSD1 can de-repress genes involved in differentiation and cell cycle arrest, curbing breast cancer cell growth and potentially resensitizing tumors to hormonal or chemotherapies. Several LSD1 inhibitors (some in clinical trials for other malignancies) are being evaluated in breast cancer models, and there is growing evidence that combining an LSD1 inhibitor with an HDAC or DNMT inhibitor produces a synergistic anti-cancer effect by concurrently reversing multiple epigenetic abnormalities [39].

In summary, therapeutic interventions targeting histone-modifying enzymes represent a new frontier in breast cancer treatment. By modulating histone modification status, these approaches aim to reactivate silenced tumor suppressor pathways and dampen oncogenic signals.

### Therapeutic implications of epigenetic regulation

Targeting the epigenetic landscape in breast cancer offers a promising therapeutic strategy, particularly for overcoming drug resistance. Epigenetic therapies, such as DNMT and HDAC inhibitors, are being actively studied in clinical trials, both as monotherapies and in combination with standard chemotherapy, endocrine therapy, and immunotherapy. These agents have the potential to restore normal gene expression patterns, reverse therapy resistance, and enhance the immune response in breast cancer patients [40].

Additionally, epigenetic modifications play a critical role in the development of immune evasion mechanisms in breast cancer. By reprogramming the expression of immune-related genes, tumor cells can escape immune surveillance. Targeting epigenetic regulators may not only enhance the efficacy of immunotherapies, such as immune checkpoint inhibitors, but also broaden their applicability to patients with epigenetically driven therapy resistance [41].

In triple-negative breast cancer, DNMT inhibition is being actively explored as an immunomodulatory primer; a phase II “window” trial (NCT02957968) administering decitabine followed by pembrolizumab before standard neoadjuvant chemotherapy in HER2-negative disease (including TNBC) increased stromal TILs and PD-L1 expression and achieved pathologic complete responses in a subset, supporting the concept of epigenetic re-sensitization to immunochemotherapy [42]. Ongoing studies are extending this strategy to metastatic TNBC with oral decitabine/cedazuridine (ASTX727) plus paclitaxel and pembrolizumab (NCT05673200), further operationalizing DNMT-guided combinations.

By contrast, in endocrine-resistant HR-positive disease, the phase III E2112 study found no improvement in progression-free or overall survival when the class-I selective HDAC inhibitor entinostat was added to exemestane versus exemestane alone [43]. This study highlights some of the limitations of epigenetic therapy.

Researchers are now working on combination therapies that combine new epigenetic modulators with traditional treatments, aiming to provide more breast cancer patients with longer-lasting therapeutic effects.

## Microbiome’s influence on breast cancer through epigenetic mechanisms

### Role of microbial metabolites in epigenetic regulation

Microbial metabolites, particularly SCFAs, play a pivotal role in regulating gene expression in breast cancer cells through epigenetic mechanisms. SCFAs such as butyrate, propionate, and acetate are produced by gut bacteria through the fermentation of dietary fibers and exert significant biological effects, particularly by inhibiting HDACs.

Butyrate is a potent HDACi which exerts its effect by preventing the removal of acetyl groups from histone proteins, thereby maintaining chromatin in a more open, transcriptionally active state. In breast cancer cells, butyrate has been shown to increase acetylation at key histone residues such as H3K27, promoting the expression of genes involved in cell cycle arrest and apoptosis [44]. This acetylation enhances the transcription of tumor suppressor genes, such as p21, leading to inhibited cell proliferation and increased cancer cell death [45].

Moreover, propionate and acetate are also involved in epigenetic regulation through their conversion to acetyl-CoA, a key substrate for histone acetylation [46]. The availability of acetyl-CoA influences the acetylation status of histones, particularly H3K9 and H3K56, which are involved in activating metabolic pathways essential for cancer cell survival. For example, increased histone acetylation promotes lipid biosynthesis, a process critical for cancer cell adaptation to metabolic stress such as hypoxia [47]. Thus, these SCFAs not only regulate tumor suppressor gene expression but also modify metabolic pathways that support breast cancer cell survival.

### Association of specific microbes with epigenetic modifications

Several bacteria have been linked to epigenetic changes in breast cancer. Notably, enterotoxigenic Bacteroides fragilis (ETBF) secretes a metalloprotease toxin (BFT) that profoundly remodels the breast cancer epigenome. In cell and animal models, brief exposure to BFT induces long-lasting DNA hypermethylation of multiple tumor suppressor gene (TSG) promoters (e.g. NF2, FAT4, RSK3, DCN, DOK2). This methylation silences TSG expression and promotes invasive phenotypes. Mechanistically, BFT-treated breast cells show increased DNMT and HDAC activity; treating these cells with DNMT inhibitors (5-Aza) or HDAC inhibitors (trichostatin A) reactivated the silenced genes and mitigated migration and metastasis [48]. Thus, ETBF exemplifies a pro-tumor microbe that directly causes host DNA methylation to disable anti-cancer programs.

Another pro-tumorigenic bacterium, Fusobacterium nucleatum—primarily an oral commensal but also a key player in colorectal cancer, has been identified in breast tumors; 16S rRNA gene sequencing has detected its DNA in the tumor tissues of a majority of breast cancer patients, and its presence has been linked to metastasis [49]. Recent work showed that Fusobacterium nucleatum infection upregulates oncogenic microRNAs in breast cancer cells: specifically, it induces host miR-21-3p, which targets and suppresses the transcription factor FOXO3. FOXO3 normally activates cell-cycle arrest and apoptosis genes, so its downregulation via miR-21 drives EMT and migration. In vivo, Fusobacterium nucleatum–infected tumors have more metastases, an effect reversed by inhibiting miR-21 [50]. This is an epigenetic mechanism in the sense that the bacterium alters a post-transcriptional gene silencer (miR-21) to reprogram the host transcriptome. Beyond microRNAs, intratumoral Fusobacterium nucleatum can reprogram the epitranscriptome: infection elevates METTL3 (observed also in breast cancer cell lines), deposits m6A on the c-MYC 3′UTR, and via YTHDF1 stabilizes c-MYC to drive proliferation and metastasis [51].

In contrast, some commensal bacteria are anti-tumorigenic via epigenetic routes. Probiotic genera (Lactobacillus, Bifidobacterium, Akkermansia) tend to enhance anti-cancer epigenetic programs. For instance, Lactobacillus spp. can produce lactic acid and promote immune modulation, and Bifidobacterium produces folate (enhancing DNA methylation of tumor suppressors). These bacteria also increase SCFA levels systemically, which as above inhibit HDACs and thus favor tumor-suppressor expression. Studies in mice show that supplementing Lactobacillus plantarum increases gut butyrate producers and elevates colonic SCFAs [52]; analogous effects in the breast could amplify histone acetylation. Although specific epigenetic targets in breast remain to be fully charted, probiotic treatment often correlates with upregulation of p21, p53 and downregulation of inflammatory miRNAs in tumors, consistent with enhanced chromatin accessibility at protective loci.

Another example is Akkermansia muciniphila, a gut mucin-degrader that appears enriched in responders to certain breast cancer therapies [20, 53]. Akkermansia stimulates host STING-type I interferon signaling in breast tumors (enhancing immune surveillance), which may in turn recruit chromatin modifiers (e.g. histone acetyltransferases at IFN gene loci) [54]. Conversely, dysbiosis-linked bacteria such as Escherichia coli, some strains of which produce colibactin, are known to induce DNA damage and inflammatory signaling, potentially leading to aberrant methylation of damage-response genes [55].

In summary, specific bacterial species have been tied to epigenomic remodeling in breast cancer. Pathogenic bacteria (ETBF, Fusobacterium nucleatum, Gram-negative pathobionts) tend to trigger signaling cascades (e.g. TLR/MyD88/NF-κB, WNT/YAP) that recruit DNA methyltransferases or microRNAs to silence tumor suppressors [48, 50]. Beneficial commensals (butyrate producers, probiotics) supply metabolites that inhibit HDACs and support methyl-donor availability, thereby maintaining an open chromatin state at growth-inhibitory genes [56]. These mechanistic insights, from the interactions between microbial enzymes and toxins to host epigenetic modifications, elucidate how the microbiome is both a driver and a therapeutic target for breast cancer.

We synthesized the last decade of evidence on microbiota–epigenome interactions relevant to breast cancer across human, animal and in vitro studies, and stratified findings by microbial role and source (Table 1). Direct epigenetic evidence specific to breast cancer was prioritized; entries supported mainly by cross-cancer or immune-epigenetic data are explicitly flagged as “Comparative evidence” to avoid over-extrapolation and to highlight targets for future validation.

**Table 1:** Microbiota implicated in breast cancer: epigenetic mechanisms and cancer-related outcomes

Microbe/community	Beneficial/harmful	Epigenetic effects	Breast cancer outcomes (model/population)	Context (gut vs intratumoral)	References
Enterotoxigenic Bacteroides fragilis (ETBF)	Harmful	BFT drives promoter DNA hypermethylation & histone deacetylation of TSGs; demethylating agents/HDACi reverse	Promotes migration/invasion, stemness & chemoresistance (in vitro/animal); detected in malignant breast tissue in recent work	Gut (can be intratumoral)	[48, 57]
Fusobacterium nucleatum	Harmful	Upregulates miR-21-3p to suppress FOXO3 axis	Increases migration/metastatic potential (in vitro/animal)	Intratumoral (may originate from oral/gut)	[50]
Escherichia coli (pks + , colibactin)/Enterobacteriaceae	Harmful	Colibactin causes DNA double-strand breaks → durable genome/epigenome remodeling (cross-cancer mechanism)	Enterobacteriaceae/E. coli enrichment in breast tumor tissue; (direct BC epigenetic proof limited)	Intratumoral (likely translocated from gut)	[58, 59]
Butyrate producers (e.g., Faecalibacterium prausnitzii)	Beneficial	Butyrate is an endogenous HDAC inhibitor → histone hyperacetylation; miRNA modulation	Reduced in BC patients (gut); butyrate induces apoptosis/inhibits proliferation in BC cell lines	Gut	[60, 61]
Akkermansia muciniphila	Beneficial	Produces SCFAs (propionate) that can mediate HDAC inhibition/histone acetylation (indirect)	Suppresses tumor growth & reverses stress-induced progression in BC mouse models	Gut	[62, 63]
Bifidobacterium (e.g., B. longum)	Beneficial	Promotes FOXP3 promoter demethylation & Treg induction in immune models (immuno-epigenetics)	In BC survivors, probiotic mixes improved gut diversity; (direct BC epigenetic links are scarce)	Gut	[64, 65]

### Impact of the microbiome on treatment sensitivity

**Fig. 2:**
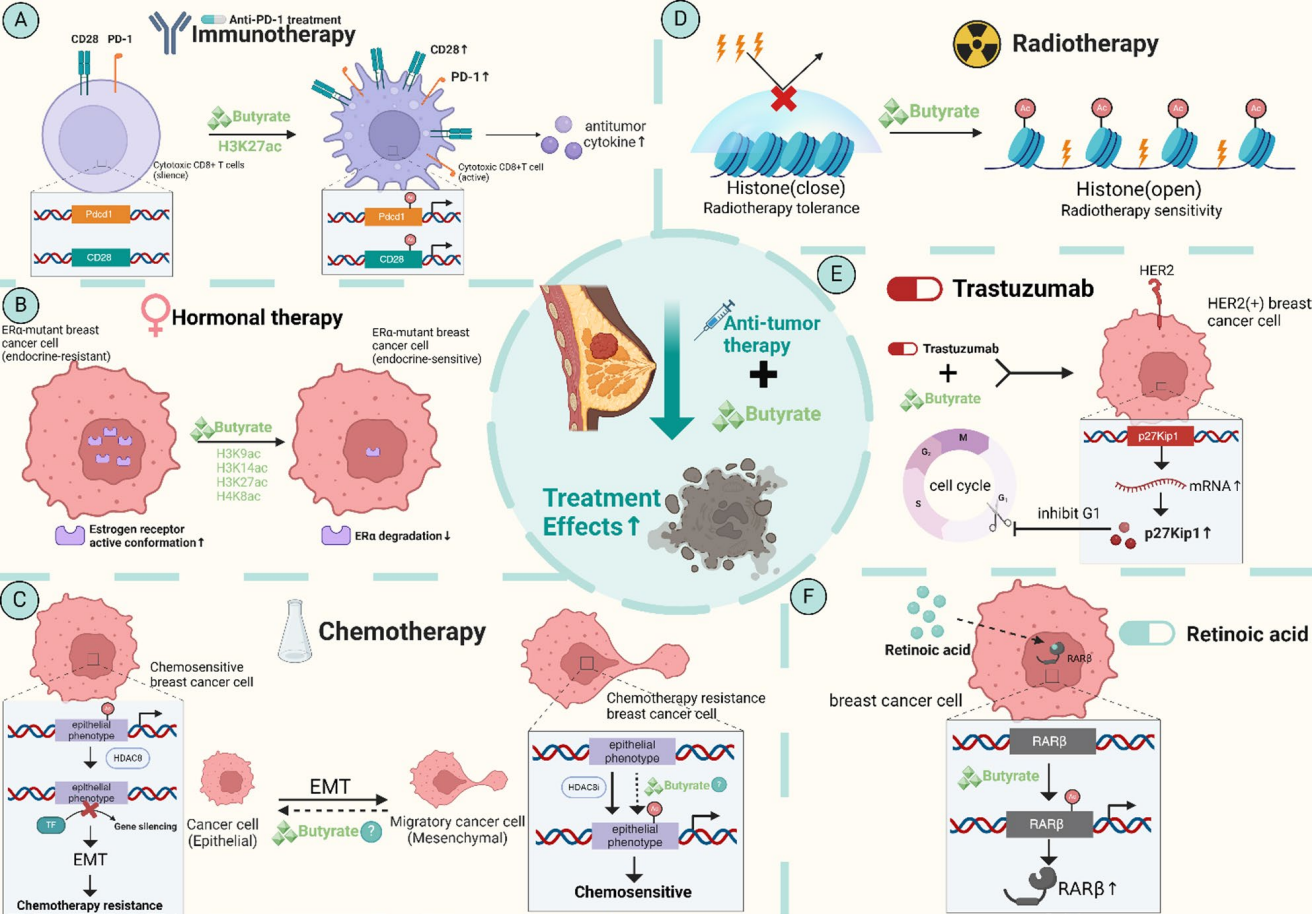
The impact of butyrate on breast tumor treatment. Epigenetic effects of microbial short-chain fatty acids (butyrate) on breast cancer treatment. The figure is divided into panels A–F illustrating the following: **A** Sodium butyrate enhances H3K27 acetylation (H3K27ac) at the promoter regions of Pdcd1 and Cd28 in CD8 + T cells, thereby promoting the expression of PD-1 and CD28, which enhances the efficacy of anti-PD-1 therapy. **B** Mutant ERα (e.g., D538G and Y537S) leads to constitutive activation, causing resistance to endocrine therapies like tamoxifen and fulvestrant. Research indicates that sodium butyrate promotes the degradation of ERα through its HDAC inhibitory activity, thereby restoring sensitivity to endocrine treatment. **C** Epithelial-to-mesenchymal transition (EMT) is a critical process that facilitates cancer cell migration and chemotherapy resistance, marked by the loss of E-cadherin. The EMT process involves the transcription factor TWIST, which recruits HDAC-containing complexes to inhibit E-cadherin expression. Furthermore, chemotherapy results in a significant loss of H3K27ac at key regulatory regions of epithelial phenotype transcription factors. However, studies suggest that impaired HDAC8 activity can restore levels of these critical transcription factors, increasing tumor sensitivity to chemotherapy. Thus, we hypothesize that sodium butyrate, as an HDAC8 inhibitor, has the potential to regulate EMT, supported by similar studies in ovarian cancer. **D** The overall compaction level of chromatin is an important mechanism influencing radiation sensitivity. Sodium butyrate can alter chromatin structure through its HDAC inhibitory activity, thereby enhancing the radiation sensitivity of breast cancer cells. **E** The combination of sodium butyrate and trastuzumab can enhance the growth-inhibitory effects on HER2-overexpressing breast cancer cells, potentially associated with increased mRNA and protein levels of the cyclin-dependent kinase inhibitor p27Kip1. **F** Retinoic acid regulates gene expression through the activation of retinoic acid receptors (RAR) and retinoid X receptors (RXR). Dysregulation of retinol metabolism is observed in breast cancer cells, where RARβ may serve as a molecular target for sodium butyrate. By upregulating RARβ through its HDAC inhibitory activity, sodium butyrate—when used in conjunction with retinoic acid in breast cancers with epigenetic silencing of RARβ—can enhance anti-cancer effects (Figure created by the authors using BioRender)

The microbiome’s role in influencing breast cancer treatment responses, particularly through epigenetic pathways, is an area of active investigation. To orient the reader, Fig. 2 schematizes how microbiome-derived short-chain fatty acids, with particular focus on butyrate, reprogram tumor and immune epigenetics to shape therapy response in breast cancer.

Immunotherapy, such as anti-PD-1 therapy, has shown promise in treating various cancers, including breast cancer. However, the efficacy of these therapies is often dependent on the patient’s microbiome composition. Figure 2A highlights an immunotherapy route: butyrate, by acting as an HDAC inhibitor, enhances the TCR signaling of cytotoxic CD8 + T cells. Mechanistically, butyrate increases H3K27ac at the PD-1 (Pdcd1) and CD28 promoters in human CD8⁺ T cells, upregulates these loci. This effect sensitizes T cells to anti-PD-1 therapy, thereby enhancing the efficacy of immunotherapies aimed at reactivating T cells to target breast cancer cells [18].

Consistent with Fig. 2B, Schoeller et al. demonstrate that SCFAs display selective estrogen receptor downregulator like activity in ER⁺ breast cancer: butyrate and propionate induce ERα downregulation in MCF-7/T47D cells expressing wild-type or ESR1 mutants (Y537S/D538G), in parallel with increased histone acetylation indicative of HDAC inhibition; pan-HDAC inhibitors (e.g., vorinostat, entinostat, panobinostat) similarly reduce ERα,. These data provide a mechanistic basis for our Fig. 2B model in which sodium butyrate as a HDAC inhibitor, depletes wild-type and mutant ERα pools and thereby counteracts endocrine resistance, potentially restoring sensitivity to antiestrogens such as tamoxifen or fulvestrant [66].

Panel C illustrates that EMT-linked therapy resistance in breast cancer is associated with HDAC-containing repressor complexes and chemotherapy-induced chromatin changes. Mechanistically, the EMT driver TWIST recruits the Mi2/NuRD–HDAC2 complex to the E-cadherin (CDH1) promoter to enforce transcriptional repression and metastatic behavior, a deacetylase activity that is suppressible by sodium butyrate [67]. In basal-like models exposed to chemotherapy, HDAC8 is upregulated; ChIP-seq shows loss of H3K27ac at master epithelial transcription factors, while HDAC8 inhibition restores these epithelial TFs and re-sensitizes tumor cells to cytotoxic agents [68]. Clinically, HDAC8 is increasingly recognized as an oncogenic, targetable epigenetic node in breast cancer, and HDAC8-active inhibitors including sodium butyrate are under consideration [69]. Functionally supporting this axis, sodium butyrate enhances E-cadherin expression and chemosensitivity (e.g., to cisplatin) in resistant carcinoma models partly reversing EMT features, even though full phenotypic reversion may not occur [70].

In Fig. 2D, Cho et al. showed that the HDAC inhibitor sodium butyrate increases radiosensitivity in MCF-7 breast cancer cells, and that combining sodium butyrate with the DNA demethylating agent 5-aza-2′-deoxycytidine further potentiates the effect: in MCF-7, post 6 Gy survival fell from 87% (radiotherapy alone) to 55.7% (radiotherapy + sodium butyrate) and 45.7% (sodium butyrate + 5-aza-DC + RT). This aligns with an epigenetic radiosensitization model in which HDAC inhibition relaxes chromatin (increasing acetylation) while 5-aza-DC demethylates tumor suppressor loci. It is noteworthy that sodium butyrate alone does not alter the DNA methylation state, supporting the view that the sodium butyrate-mediated radiosensitization mechanism is chromatin compaction rather than demethylation [71].

In line with Fig. 2E, Chen et al. report that combining sodium butyrate with trastuzumab produces greater growth inhibition in HER2 overexpressing SKBR3 breast cancer cells than either agent alone, accompanied by increased cyclin-dependent kinase inhibitor p27Kip1 mRNA and protein, which may be related to the HDAC inhibitor properties of sodium butyrate; notably, this effect is absent in HER2 negative HCC1937 cells, underscoring HER2 dependence. These findings support our model that HDAC inhibition by sodium butyrate can potentiate HER2 targeted therapy via cell-cycle restraint in HER2 overexpressing breast cancer [72].

Finally, consistent with Fig. 2F, Andrade et al. show that sodium butyrate, a dietary HDAC inhibitor, potentiates retinoid signaling in ER positive MCF-7 cells: sodium butyrate inhibits proliferation and, when combined with vitamin A, yields greater growth inhibition than either agent alone. Mechanistically, butyrate robustly elevates RARβ mRNA, positioning RARβ as a butyrate-responsive target and providing a rationale for butyrate-retinoid cotreatment, particularly in breast cancers with epigenetic silencing of RARβ [73].

Furthermore, the gut microbiome has been shown to influence the response to chemotherapy and radiotherapy in breast cancer. Patients with a diverse and balanced microbiome, rich in SCFA-producing bacteria, tend to respond better to these treatments. SCFAs have been found to enhance the sensitivity of cancer cells to chemotherapy and radiotherapy by promoting histone acetylation and altering DNA methylation patterns that affect cell survival pathways [74]. In contrast, dysbiosis, characterized by a reduction in beneficial microbial species, is associated with increased treatment resistance. The loss of SCFA production, for example, diminishes the anti-tumor immune response and reduces the effectiveness of cancer therapies [75].

Given the strong link between microbiome composition and treatment response, strategies such as probiotics, prebiotics, and fecal microbiota transplantation (FMT) are being explored as potential adjunct therapies to restore a healthy microbiome and improve treatment outcomes in breast cancer patients. By modulating the microbiome and its influence on the epigenetic landscape, these interventions may enhance the efficacy of existing treatments, including chemotherapy, radiotherapy, and immunotherapy [20–22]. This interplay between the microbiome and epigenetics offers promising avenues for novel therapeutic strategies aimed at overcoming resistance and improving the prognosis for breast cancer patients.

## Microbiome and metabolic diseases in relation to breast cancer

### Impact of metabolic diseases on breast cancer

Metabolic diseases, particularly obesity and type 2 diabetes mellitus (T2DM), have been strongly associated with an increased risk of breast cancer. These diseases are not only linked to breast cancer incidence but also contribute to more aggressive tumor phenotypes and poorer prognosis. Obesity and T2DM both lead to systemic metabolic alterations, such as insulin resistance, hyperinsulinemia, chronic inflammation, and altered adipokine secretion, which create a pro-tumorigenic environment that can promote breast cancer development and progression [76].

(1) Obesity and Breast Cancer: Obesity is a well-established risk factor for postmenopausal breast cancer, as it induces chronic low-grade inflammation and increases the production of adipokines such as leptin and resistin, while reducing levels of adiponectin [77]. These changes alter insulin sensitivity and promote the activation of oncogenic pathways. Elevated leptin levels, for instance, have been shown to stimulate breast cancer cell proliferation via the JAK/STAT, PI3K/AKT, and MAPK signaling pathways [78]. Furthermore, adipose tissue in obese individuals serves as a significant source of estrogen production through the aromatization of androgens, contributing to the development of ER + breast cancer [79].

Obesity also increases the expression of inflammatory mediators such as TNF-α, IL-6, and CRP, which are associated with tumor initiation, growth, and metastasis. These inflammatory cytokines promote epigenetic changes, including DNA methylation and histone modifications, that regulate gene expression in breast cancer cells. For example, obesity-induced inflammation has been linked to the hypermethylation and silencing of tumor suppressor genes such as BRCA1 and PTEN, which accelerates tumorigenesis [80].

(2) Diabetes and Breast Cancer: T2DM is associated with increased breast cancer risk, particularly in postmenopausal women. Hyperinsulinemia, a hallmark of T2DM, enhances insulin signaling through the insulin receptor (IR) and IGF-1 receptor (IGF-1R) pathways, promoting cell proliferation and survival. Insulin resistance in diabetic patients leads to compensatory hyperinsulinemia, which activates downstream signaling molecules such as PI3K/AKT and Ras/MAPK, both of which are implicated in breast cancer progression [81].

In addition, hyperglycemia and insulin resistance contribute to oxidative stress and the production of advanced glycation end-products (AGEs), which bind to the RAGE (receptor for advanced glycation end-products) on breast cancer cells. This interaction upregulates GADD45α, which in turn triggers DNA demethylation of the MMP-9 promoter, thereby promoting metastasis [82]. The metabolic dysregulation observed in T2DM also induces epigenetic alterations, including changes in DNA methylation and miRNA expression, that influence breast cancer development. For instance, hyperglycemia has been shown to increase the expression of miR-21, an oncogenic miRNA, which suppresses tumor suppressor genes such as PTEN and enhances cancer cell proliferation [83].

### Role of the microbiome in metabolic diseases and breast cancer progression

The gut microbiome plays a critical role in the regulation of metabolic diseases such as obesity and T2DM, which in turn influence breast cancer risk and progression. Dysbiosis, or the imbalance of gut microbial communities, is commonly observed in both obesity and diabetes and is thought to contribute to systemic inflammation, insulin resistance, and altered lipid metabolism—all of which are key factors in breast cancer development.

**Fig. 3:**
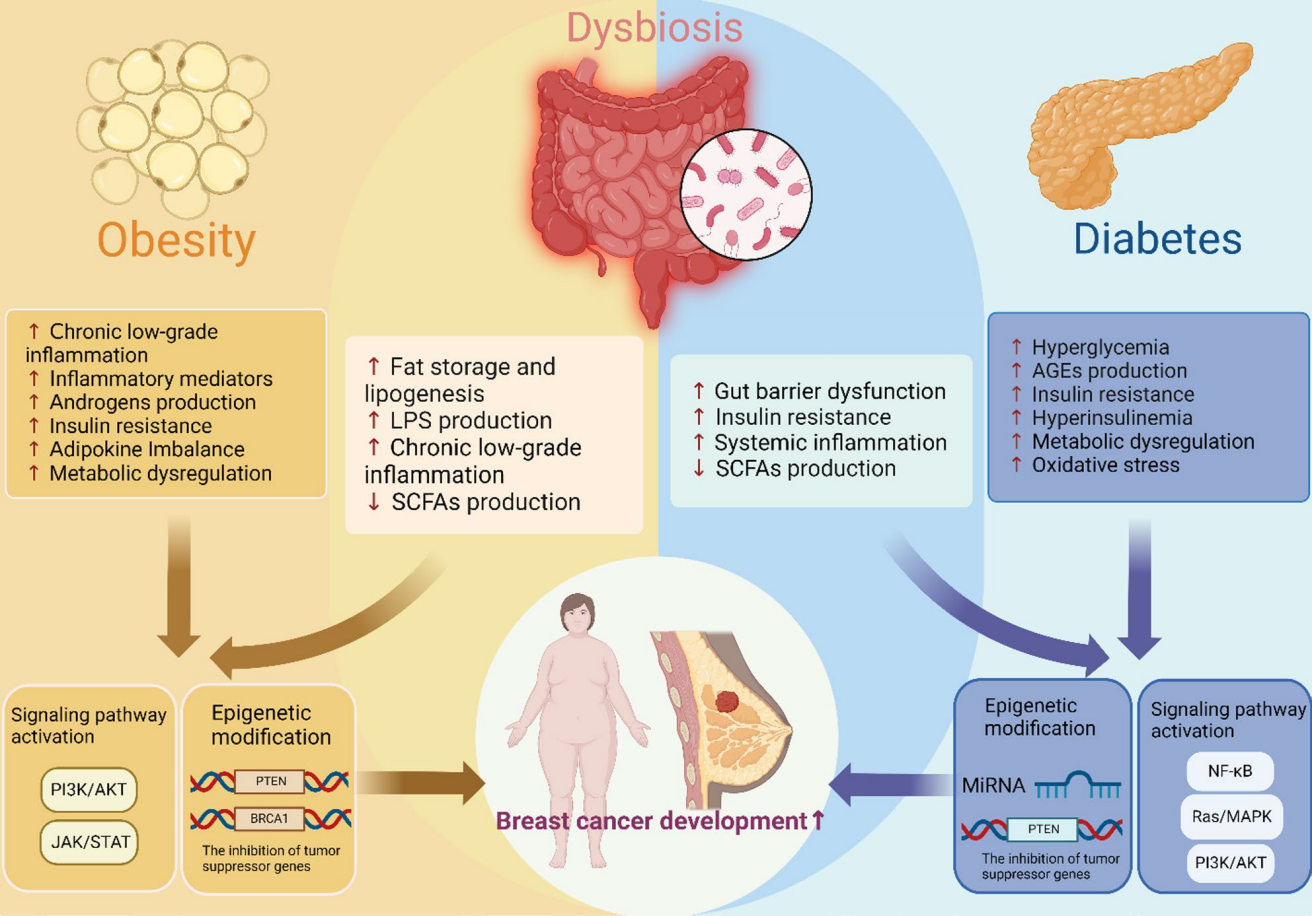
The role of metabolic diseases and the microbiome in breast cancer. Diagram illustrating the interplay between metabolic diseases, the gut microbiome, and breast cancer. Obesity-associated gut microbiome changes (Firmicutes, Bacteroidetes) lead to elevated bacterial lipopolysaccharide levels and chronic inflammation. These factors promote hypermethylation and silencing of tumor suppressor genes (e.g., BRCA1, PTEN), thereby accelerating breast cancer development. In T2DM, a decrease in butyrate-producing bacteria and an increase in pro-inflammatory Proteobacteria compromise the intestinal barrier and increase systemic inflammation and insulin resistance. Meanwhile, T2DM induces epigenetic changes, such as DNA methylation of certain tumor suppressor genes and upregulation of oncogenic microRNAs (e.g., miR-21), ultimately facilitating breast cancer progression and metastasis (Figure created by the authors using BioRender)

To orient the reader, Fig. 3 schematically integrates these metabolic drivers with gut-microbiome-derived signals (dysbiosis, barrier dysfunction lipopolysaccharides (LPS) metabolic endotoxemia and shifts in short-chain fatty acids) that converge on inflammatory and epigenetic pathways relevant to breast cancer.

(1) Microbiome and Obesity: Obesity is associated with alterations in the composition of the gut microbiome, with an increase in Firmicutes and a decrease in Bacteroidetes. This shift in microbial balance enhances the ability of the microbiome to extract energy from the diet, leading to increased lipogenesis and fat storage [84]. In addition to altering energy metabolism, the obese microbiome produces microbial metabolites, such as LPS, that contribute to metabolic endotoxemia, a condition characterized by increased circulating LPS levels and chronic low-grade inflammation. This inflammatory state is linked to breast cancer development through the activation of inflammatory pathways and the promotion of epigenetic modifications that alter gene expression [85].

Furthermore, the gut microbiome influences the production of SCFAs, which have epigenetic effects on host cells. In obese individuals, the reduced production of butyrate, an SCFA with anti-inflammatory properties, may contribute to a pro-tumorigenic environment. Butyrate is known to act as an HDAC inhibitor, promoting histone acetylation and the activation of tumor suppressor genes. The dysregulation of SCFA production in obesity may therefore exacerbate breast cancer risk by diminishing the epigenetic regulation of genes involved in tumor suppression [86].

(2) Microbiome and Diabetes: In T2DM, gut dysbiosis is characterized by a reduction in butyrate-producing bacteria and an increase in pro-inflammatory species such as Proteobacteria and Bacteroides [87]. The reduction in butyrate and other SCFAs compromises the integrity of the intestinal barrier, leading to increased gut permeability and the translocation of microbial products such as LPS into the circulation. This metabolic endotoxemia triggers systemic inflammation and insulin resistance, both of which are associated with an increased risk of breast cancer [85].

The gut microbiome also influences epigenetic regulation in T2DM by modulating DNA methylation patterns and miRNA expression. For example, dysbiosis in diabetic individuals has been linked to the hypomethylation of genes involved in insulin signaling pathways, which exacerbates hyperinsulinemia [88]. Additionally, microbial metabolites, such as secondary bile acids, have been shown to influence histone modifications that regulate gene expression in both metabolic and cancer-related pathways [89].

The interplay between the gut microbiome, metabolic diseases, and breast cancer highlights the potential of microbiome-targeted therapies as a means of reducing cancer risk in patients with obesity and diabetes. Probiotics, prebiotics, and dietary interventions aimed at restoring a healthy gut microbiome may help to modulate epigenetic changes associated with these metabolic conditions, thereby reducing the risk of breast cancer progression. Moreover, interventions that increase the production of beneficial SCFAs, such as butyrate, could enhance the epigenetic regulation of tumor suppressor genes and improve outcomes in breast cancer patients.

## Future therapeutic strategies for breast cancer based on the microbiome

### Microbiome modulation and therapeutic prospects

The modulation of the gut microbiome represents a promising avenue for novel therapeutic strategies in the treatment of breast cancer. By influencing key metabolic and immune pathways, the gut microbiome exerts significant control over cancer progression and the patient’s response to treatment. The use of probiotics, dietary interventions, and FMT are emerging strategies aimed at restoring a healthy microbial balance, which may offer substantial benefits in cancer management.

1. Probiotics: The administration of probiotics, particularly strains of Lactobacillus and Bifidobacterium, has been shown to modulate the gut microbiome and improve outcomes in breast cancer. These beneficial bacteria enhance the production of SCFAs, such as butyrate, which act as HDAC inhibitors, promoting the acetylation of tumor suppressor gene promoters and leading to the activation of anti-tumorigenic pathways. Additionally, probiotics help to suppress inflammation and strengthen the intestinal barrier, reducing systemic inflammation, a key driver of breast cancer progression.
2. Dietary Interventions: Dietary modification is another important tool for modulating the gut microbiome. Diets rich in fiber promote the growth of SCFA-producing bacteria, particularly butyrate producers, which are known to exert epigenetic effects by inhibiting HDACs and promoting histone acetylation, leading to the reactivation of tumor suppressor genes [90]. For example, Mediterranean diets, high in plant-based foods, have been linked to increased microbiome diversity and improved outcomes in cancer patients [91]. Furthermore, diets rich in polyphenols (e.g., from fruits and vegetables) can enhance the growth of beneficial microbial species and exert anti-inflammatory effects, further reducing breast cancer risk [92]. This article summarizes the relationship between dietary substances related to changes in SCFA production and their effects on the microbiome, as shown in Table 2. The dietary substances and their microbial metabolites related to the epigenetic regulation of breast cancer are shown in Table 3.
3. Fecal Microbiota Transplantation: FMT, a technique that involves the transplantation of stool from a healthy donor into a patient, has demonstrated potential in re-establishing a balanced microbiome and modulating the immune system [113]. Although its application in breast cancer is still under investigation, FMT has shown promise in restoring the efficacy of immunotherapies in other cancers by enhancing the immune response. By rebalancing the gut microbiota, FMT may reduce therapy resistance and improve the response to treatments such as immunotherapy and chemotherapy in breast cancer [114].

**Table 2:** Interaction between dietary substances and gut microbiota

Dietary substance	Impact on Gut microbiota	Increased SCFA-producing microbiota	Microbial metabolites	Metabolism-involved bacteria	References
Soy: Genistein	Increased: Lactococcus, Akkermansia muciniphila, Eubacterium dolichum	Family Lachnospiraceae and Ruminococcaceae	Equol: Increased affinity to estrogen receptor β, dihydrogenistein, 4-ethylphenol	Eggerthella species, Lactobacillus species	[93]
	Decreased: Bacteroides eggerthii, Bacteroides uniformis, Paraprevotella, Anaerostipes, Blautia, Coprobacillus				
Pomegranate, Blueberry: Ellagic Acid and Ellagitannins	Increased: Akkermansia spp.	Family Lachnospiraceae and Ruminococcaceae	Urolithins	Gordonibacter species, Ellagibacter species, Enterocloster species	[94, 95]
	Decreased: Order Bacteroidales				
Cruciferous Vegetables: Glucosinolates	Increased: Bacteroidetes, Bacteroides genus	Bacteroides vulgatus, Bacteroides thetaiotaomicron	Sulforaphane (SFN), formed when plant enzymes are degraded during cooking or processing	Bacteroides thetaiotaomicron, Escherichia coli, Enterococcus casseliflavus, Enterococcus gallinarum	[96, 97]
	Decreased:Firmicutes				
Green Tea: Green Tea Catechins (GTC)	Increased: Bifidobacterium spp., Lactobacillus spp., Enterococcus spp.	Faecalibacterium, Roseburia, Blautia, Eubacterium, Bifidobacterium, Coprococcus	3,4-dihydroxyphenyl-γ-valerolactone, 4-hydroxy-5-(3',4'-dihydroxyphenyl)	Firmicutes, Actinobacteria	[98, 99]
	Decreased: Bacteroides, Prevotella, Clostridium histolyticum, Clostridium coccoides				
Grapes, Berries, and Red Wine: Resveratrol (Res)	Increased: Lactobacillus reuteri, Lactobacillus intestinalis	Lactobacillus reuteri, Lactobacillus intestinalis	Resveratrol sulfate (RES-sulfate), Resveratrol glucuronide (RES-glucuronide), dihydroresveratrol (DHR)	Ligilactobacillus salivarius, Lactobacillus spp., Bifidobacterium?, Rikenellaceae?	[100, 101]
	Decreased: Blautia, Bacteroides caecimuris, Bacteroides satorii, Ruminococcaceae, Parabacteroides, Parasutterella

**Table 3:** The epigenetic regulatory effects of dietary substances and their metabolites on breast cancer

Dietary substances or their microbial metabolites	Breast cancer cell line	The role of enzymes related to epigenetic regulation	Effects on tumor cells	References
Genistein	BCM-3204 (Tamoxifen-resistant)	Inhibit DNMT3b, HDAC2, Tet3	By altering the expression levels of epigenetic-related genes, the reactivation of tumor suppressor genes can be promoted	[102]
	MCF-7, MDA-MB-231	Down-regulation DNMT1, DNMT3a, DNMT3b	Increased the mRNA expression levels of tumor suppressor genes such as ATM, APC, PTEN, and SERPINB5, leading to the inhibition of proliferation and induction of apoptosis in breast cancer cells	[103, 104]
		Down-regulation HDAC1, HDAC6	Promoted the expression of tumor suppressor genes (such as p16 and p21)	[104]
		Down-regulation EZH2, SUV39H1	Inhibited histone modifications associated with gene silencing (such as H3K27me3 and H3K9me3), reducing the expression of tumor-promoting genes (such as BMI1 and c-MYC)	[104]
	SHR (fully transformed breast cancer cells) and SH (precancerous cells)	Activate HMTs	Promoting H3K4me3 in the promoter regions of p16 and p21 enhances the expression of tumor suppressor genes. GE inhibits the two histone modifications associated with gene silencing, H3K9me3 and H3K27me3, further reducing the expression of oncogenes such as BMI1 and c-MYC	[105]
	SHR	Inhibit HDAC	The expression of tumor suppressor genes (such as p16 and p21) increases, thereby inhibiting cell proliferation and inducing apoptosis	[105]
Tannic acid	MCF-7	Inhibit DNMT1, HAT	Inhibit the proliferation of breast cancer cells and induce apoptosis by increasing the expression of apoptosis-related genes such as Bax and Caspase	[106, 107]
UA (Ursolic Acid)	MCF-7, MDA-MB-231	Down-regulation of DNMT1, DNMT3a,HDAC1,HDAC4,HDAC6	DNMT↓: The re-expression of tumor suppressor genes such as p53 and p21 leads to the inhibition of cell proliferation and the induction of apoptosis	[106, 107]
			HDAC↓: Promotes the activation of tumor suppressor genes such as Bax and p21, thereby inducing cell cycle arrest and apoptosis. Especially in MDA-MB-231 cells, UA significantly inhibited its invasive ability and migration	[106, 107]
SFN	MCF-7, MDA-MB-231	Inhibit DNMT1,DNMT3a, DNMT3b	Acting on the promoter regions of tumor suppressor genes such as p21 and PTEN, it reactivates tumor suppressor genes. It also reduces the DNA methylation levels of telomerase reverse transcriptase (hTERT), decreasing hTERT activity and inhibiting the ability for unlimited proliferation	[104, 106, 108]
		Inhibit HDAC1, HDAC4, HDAC6, HDAC11	Re-express tumor suppressor genes (such as p21 and KLOTHO)	[104, 106, 108]
		Inhibit HMT:EZH2,SUV39H1	Reduced histone modifications associated with gene silencing, such as H3K27me3 and H3K9me3, leading to the reactivation of tumor suppressor genes	[104, 108]
	HER2/neu mouse model	Inhibit HDAC1, DNMT1, DNMT3a, DNMT3b	HDAC1↓: Reactivation of tumor suppressor genes (such as p16 and p21)	[109]
EGCG (Epigallocatechin-3-Gallate)	MCF-7, MDA-MB-231	Inhibit DNMT1,DNMT3a, DNMT3b	Reactivating the expression of the tumor suppressor gene SCUBE2 in its promoter region	[110]
GTP (Green Tea Polyphenols)	HER2/neu mouse model	Inhibit DNMT1, DNMT3a, DNMT3b	Reactivating the tumor suppressor genes PTEN and p53	[109]
RES	MCF-7, MDA-MB-231	Inhibit HDACs, DNMT1, DNMT3a, DNMT3b	Upregulated the expression of the pro-apoptotic gene Bax and downregulated the expression of the anti-apoptotic gene Bcl-2	[111]
	MCF-7, MCF10CA1a	(Works by enhancing remethylation and chromatin remodeling to silence upstream genes in oncogenic pathways)	Induces increased DNA methylation in enhancers of key regulatory genes such as GLI2 and WNT4, accompanied by increased H3K27me3, decreased H3K27ac/H3K9ac, and loss of transcription factor binding, thereby causing the Hedgehog and Wnt pathways to shift from open to heterochromatinized silencing, and consequently downregulating downstream oncogenes such as BCL2, EpCAM, CCND1, and CYR61	[112]

### Microbiome-assisted personalized therapy

Personalized treatment strategies that integrate microbiome modulation are gaining traction as a way to optimize breast cancer therapy. The gut microbiome influences the metabolism of drugs, the efficacy of cancer treatments, and the patient’s immune response, which makes it an important consideration in developing tailored therapeutic approaches. Research shows that FMT may thus improve the effectiveness of immunotherapy in breast cancer patients [114]. Personalized microbiome modulation strategies offer the potential to improve treatment outcomes by tailoring interventions to the specific microbial composition of each patient. This approach may include probiotic supplementation, dietary adjustments, or FMT to restore beneficial microbial populations and optimize the patient's response to standard therapies.

### Epigenetic regulation therapy targeting microbes

The interplay between the microbiome and epigenetic regulation opens avenues for targeted strategies that reshape the epigenetic landscape of breast cancer cells. Emerging evidence links intestinal and mammary microbiota with DNA methylation and histone modifications relevant to breast tumor biology.

Epigenetic agents—including DNMT inhibitors and HDAC inhibitors—can reverse aberrant gene silencing and re-engage tumor-suppressive programs, although their clinical performance in breast cancer has been mixed and remains under active investigation. Recent trial mapping highlights ongoing combination strategies with HDAC inhibitors in breast cancer, whereas DNMT inhibitors have yet to demonstrate consistent clinical benefit in this disease setting.

Mechanistically, microbiome-derived metabolites offer a biologic rationale for combined approaches: short-chain fatty acids such as butyrate function as endogenous HDAC inhibitors and can modulate chromatin accessibility and transcriptional programs in host tissues. Accordingly, pairing epigenetic agents with microbiome-directed interventions—for example, dietary fiber to augment SCFA production, defined probiotics/postbiotics, or other microbiota-modulating modalities—could assemble more comprehensive regimens that concurrently target tumor-intrinsic epigenetic states and microbe-driven signaling linked to therapy resistance, with the goal of improving response durability.

## Conclusions

In conclusion, this review highlights the critical role of the microbiome and epigenetic regulation in the development, progression, and treatment of breast cancer. The microbiome exerts significant influence on breast cancer through metabolites such as SCFAs, which modulate gene expression via epigenetic mechanisms like DNA methylation and histone acetylation. Understanding these interactions provides valuable insights into the mechanisms of tumorigenesis and therapeutic resistance. Future research should focus on elucidating the precise molecular pathways by which the microbiome influences epigenetic modifications in breast cancer and explore novel therapeutic strategies targeting both the microbiome and the epigenome. Clinically, integrating microbiome modulation with epigenetic therapies holds promise for enhancing treatment efficacy, reducing resistance, and improving patient outcomes in breast cancer management.

## Data Availability

No data was used for the research described in the article.
